# Scientific Items

**Published:** 1886-09-20

**Authors:** 


### SCIENTIFIC ITEMS.

**The Latest Microscopical Discoveries.**—The Rev. Dr. Dallinger, F. R. S., and President of the Royal Microscopical Society, in a recent lecture at Firth College, Sheffield, described the result of three years' close study of the minutest forms of life. He stated that he was now possessed of lenses so constructed as to realize results which only five years ago were declared by mathematicians to be impossible of accomplishment. By means of these he threw upon an illuminated screen various forms of minute life. One of these was a piece of hard chalk, of the size of a pin's head, which contained shells estimated to be equal to four millions in an ounce of chalk. He also showed a drop of water, taken by himself near the reservoir at Preston, Lancashire, containing specimens of desmids, which, cubically measured, were, only about one millionth of an inch. Taking a single specimen of living organism from a drop of water, he showed it upon the screen, and said, by the aid of very powerful lenses which had come into his possession only within the last few months, he had discovered this, which was the minutest organism known. He had measured the flagellum or motor fibre of this organism, and found it to be the 204,700,00th of an English inch. Dr. Dallinger subsequently gave the results of his recent researches on the subject of bacteria and putrefactive organisms, and said the work of these organisms was to break up and set free from dead organisms the elements of which they were made, so as to render them capable of circulating in new generations. In his recent labors he had found one whose duty it was to glean, as it were, the remaining particles after other forms had done their work.—*Pacific Record.*

**Danger from Umbrellas at Sea.**—In these days of electric lighting, one is often in the neighborhood of dynamos, and however short the time of exposure to their influence, pocket knives, and the steel in watches and umbrella frames, may become powerfully magnetized. On board the Princess Beatrice, the helmsman lately observed that the compass was agitated. On examination, he found that the needle was affected by the magnetized steel mounting of a parasol in the hands of a lady who was walking upon the bridge. If the lady had been at rest, so that nothing would have shown the abnormal deviation, the ship might easily have been steered out of its course, and thus been exposed to dangerous accidents.—*News and Miscellany.*

A CIGAR contains acetic, carbolic, formic, butyric, valeric, prussic, and propionic acids, also creasote, ammonia, sulphuretted hydrogen, pyridine, viridine, picoline, and rubidene, to say nothing of cabbagine and burdockic acid. That's why you can't get a good one for less than five cents.—*Ib.*

**Thunder Storms.**—From certain meteorological statistics recently published in Germany, we learn that thunder storms in that country have, during the last thirty years, been steadily increasing both in frequency and severity. The number of deaths per annum from lightning has increased in a far greater ratio than that of the increase of population. In the present state of our knowledge of the whole subject of atmospheric electricity, the cause of the phenomena of thunder storms is confessedly obscure. It is, however, very possible that some light would be thrown upon the question by a comparative study of the frequency and severity of storms during a lengthened period and over a wide geographical area.

The German *savants* incline to the opinion that the increase is to be attributed to the enormously increased production of smoke and steam which has taken place during the last three decades. But although we may admit this to be to some entent a probable *vera causa*, when we consider the very local character of thunder storms, we should naturally expect to find that it would follow that the neighborhoods of large cities, and especially of manufacturing districts, would suffer the most severely. But the statistics referred to show distinctly that the very reverse is the case. The number of storms attended by fatal results from lightning is far larger in the agricultural districts than in the towns. Upon the other hand, we ought to take into consideration the protective action of lightning conductors, with which the prominent buildings in the towns of Germany are well provided.—*Ex.*

DR. CHICHESTER A. BELL, cousin of Prof. Alexander Graham Bell, claims to have made a wonderful discovery, by means of which sound waves, which cause vibrations in a fluid jet, can be photographed and accurately reproduced by the aid of a microphone. This rival of the phonograph, Prof. Bell says, is of more importance and of greater prospective practical value than even his own invention of the telephone. The report of the things actually accomplished by this invention is marvelous.

I SAY, Gov'ner, how do yer sell ammunition?" "What's up, then? Are you going to enlist as a soldier?" "No; that's what my girl told me to get for the baby; it is sold in boxes." "Is it fuller's earth or violet powder? How are you going to use it?" "If you give me the world I couldn't tell you." "Was it magnesia?" "Hi, that's it; I am glad that you thought of that. Well, there is no difference, is there? Give us a penn-orth."—*News and Miscellany.*

A VERY complete filling for open cracks in floors may be made by thoroughly soaking newspapers in paste made of one pound of flour, three quarts of water, and a tablespoonful of alum, thoroughly boiled and mixed. Make the final mixture about as thick as putty, and it will harden like papier mache. This paper may be used for moulds for various purposes.—*Cal. Architect.*

